# Effects of Hyaluronic Acid and γ–Globulin Concentrations on the Frictional Response of Human Osteoarthritic Articular Cartilage

**DOI:** 10.1371/journal.pone.0112684

**Published:** 2014-11-26

**Authors:** Jae-Yong Park, Cong-Truyen Duong, Ashish Ranjan Sharma, Kyeong-Min Son, Mark S. Thompson, Sungchan Park, Jun-Dong Chang, Ju-Suk Nam, Seonghun Park, Sang-Soo Lee

**Affiliations:** 1 Institute for Skeletal Aging & Orthopedic Surgery, Hallym University-Chuncheon Sacred Heart Hospital, Chuncheon, Republic of Korea; 2 School of Mechanical Engineering, Pusan National University, Busan, Republic of Korea; 3 Mechanical Engineering Department, Industrial University of Ho Chi Minh City, Ho Chi Minh, Vietnam; 4 Department of Engineering Science, University of Oxford, Oxford, United Kingdom; 5 Department of Urology, University of Ulsan College of Medicine, Ulsan, Republic of Korea; Harbin Institute of Technology, China

## Abstract

Synovial fluid plays an important role in lubricating synovial joints. Its main constituents are hyaluronic acid (HA) and γ–globulin, acting as boundary lubricants for articular cartilage. The aim of the study was to demonstrate the concentration-dependent effect of HA and γ–globulin on the boundary-lubricating ability of human osteoarthritis (OA) cartilage. Normal, early and advance stage articular cartilage samples were obtained from human femoral heads and in presence of either HA or γ–globulin, cartilage frictional coefficient (µ) was measured by atomic force microscopy (AFM). In advanced stage OA, the cartilage superficial layer was observed to be completely removed and the damaged cartilage surface showed a higher µ value (∼0.409) than the normal cartilage surface (∼0.119) in PBS. Adsorbed HA and γ–globulin molecules significantly improved the frictional behavior of advanced OA cartilage, while they were ineffective for normal and early OA cartilage. In advanced-stage OA, the concentration-dependent frictional response of articular cartilage was observed with γ–globulin, but not with HA. Our result suggested that HA and γ–globulin may play a significant role in improving frictional behavior of advanced OA cartilage. During early-stage OA, though HA and γ–globulin had no effect on improving frictional behavior of cartilage, however, they might contribute to disease modifying effects of synovial fluid as observed in clinical settings.

## Introduction

Osteoarthritis (OA) is a common disease of synovial joints, characterized by the degeneration of articular cartilage that involves changes in cartilage structures and compositions [1]. The damaged cartilage surface by OA results in an increase in friction and causes partial disruption of the biphasic lubrication mechanism in articular cartilage [2]. Solid–fluid biphasic structure of cartilage is known to contribute toward lubricating diarthrodial joints through interstitial fluid pressurization [3]. Moreover, the measured frictional coefficient (µ) of articular cartilage is dependent on both the lubricants and the counterpart bearing materials used. With lubrication by synovial fluid (SF), the µ of cartilage−on−cartilage, cartilage−on−glass and cartilage−on−metal bearings are approximately ∼0.01–0.1 [4], [5], ∼0.05–0.1 [6], [7], and ∼0.2–0.5 [8], [9], respectively, depending on load bearing conditions (i.e., loading duration and magnitude). In contrast, with lubrication by individual components of SF, the µ were ∼0.03–0.3 for a cartilage−on−cartilage bearing when measured in hyaluronic acid (HA) solution [10], [11] and ∼0.2–0.4 for a cartilage−on−metal bearing in bovine serum albumin (BSA) solution [12], [13]. Also the cartilage µ is initially very small and reaches a steady-state equilibrium value due to the time-dependent decrease in cartilage interstitial pressurization [14]. Therefore, observed differences in the cartilage µ, although under the same loading conditions, are mostly caused by variation in assessment time until it reaches an equilibrium steady-state value.

SF plays an important role in lubricating synovial joints, and its main constituents are proteins (BSA and γ-globulin, 7–25 mg/mL) and HA (3–4 mg/mL) [15], acting as boundary lubricants for articular cartilage [16]. Regarding the effectiveness of HA on the lubrication of OA cartilage, evidences have been contradictory. Recent studies have shown a lower µ in HA than in Ringers solution for the surfaces of both healthy and OA bovine cartilage [17], as well as human OA cartilage in pin-on-plate tests [10]. An improved cartilage friction was observed by the penetration of HA molecules to the degenerated articular surface, but independent of HA concentration [17]. However, higher HA viscosity, due to both higher HA concentration and molecular weight, was reported to be more effective in lubricating pig OA cartilage, though HA concentration decreased with OA progression [11].

For γ-globulin which is another major protein constituent of SF, its concentration was shown to increase for patients with rheumatoid arthritis [18]. γ-globulin also showed a tendency to adsorb strongly on the rubbing surfaces with a β-sheet structure and thereby to yield large cohesive forces in a mixed or boundary lubrication regime, thus increasing the friction of poly(vinyl alcohol) hydrogel [19]. However, our previous study revealed that there was an optimal level of γ-globulin concentration for it to act as an effective boundary lubricant on the femoral head surface of Co-Cr hip prostheses [20]. Apart from reducing frictional coefficient of cartilages, constituents of SF are also responsible for biological activities of other tissues, for example glucosamine (GlcN) can regulate osteoblast differentiation and inhibit osteoclastogenesis [21], while HA has been found to possess anti-inflammatory properties [22].

Besides SF constituents, cartilage frictional behavior was also affected by the presence of superficial zone protein (SZP) at the superficial layer of articular cartilage [23]. In addition, a reduction in the SZP of bovine cartilage following trypsin digestion and secretory phospholipase A2 (sPLA2) treatments showed poor cartilage frictional behavior [23], but contradictory findings which demonstrated improvement in cartilage friction with removal of SZP was also reported [24].

For these macroscale cartilage frictional studies, however, a time–dependent increase in the µ from the initial minimum to the final equilibrium value needs to be considered [25], [26]. In contrast, microscale frictional studies by atomic force microscopy (AFM) can provide an opportunity to assess the steady state equilibrium value of cartilage µ. The absence of the fluid pressurization effect inside articular cartilage can be observed with extremely fast depletion of cartilage fluid pressure via microscale contact between an AFM tip and the cartilage surface, allowing to measure the final steady-state value according to biphasic theory [25]. Moreover, investigating the concentration–dependent cartilage lubricating ability of individual components of SF in association with OA is critical for clinically determining the treatment timing of these components for patients with OA, but there is no data available in the literature. Therefore, the purpose of this study was to examine the role of HA and γ–globulin concentrations in the boundary−lubricating ability of OA articular cartilage in human hip joints from AFM frictional measurements. Our hypothesis was that increasing concentrations of HA and γ–globulin would reduce cartilage friction.

## Materials and Methods

### Sample Preparation

**Figure 1:**
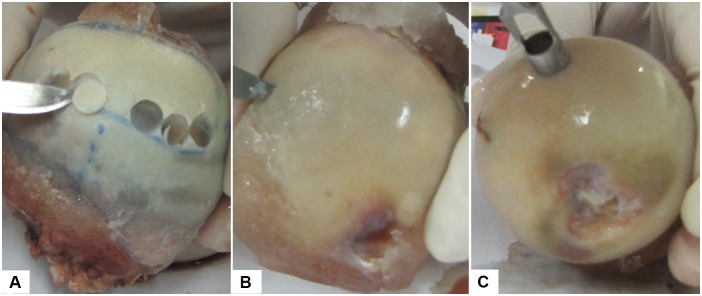
Human femoral heads at different OA stages explanted from total hip replacement operations. (A) normal cartilage, (B) early OA cartilage, and (C) advanced OA cartilage.

Three human femoral heads (34, 63, and 83 years old) with different osteoarthritis (OA) stages (i.e., each joint with a different OA stage) were explanted from total hip replacement surgery. All participants signed a written informed consent form approved by the Hallym University, Chuncheon Sacred Heart Hospital Institutional Review Board (2009–42), which also reviewed the consent form and protocol to ensure that the investigation was conducted according to the principles expressed in the Declaration of Helsinki and approved this study. The femoral heads were stored in phosphate buffered saline (PBS) solution and frozen at −20°C until being thawed for specimen preparation (Figure 1). An experienced orthopaedic surgeon visualized the femoral heads macroscopically and graded each human femoral head as per Outerbridge classification system (Table 1). Outerbridge classification can be used to garde chondral lesions with accuracy [27], [28]. Each human femoral head with grade 0, I and III has been described as normal, early, and advanced OA stage, respectively. In total, fifteen cylindrical cartilage plugs (diameter = 5 mm, height ∼0.5 mm) were sectioned from the load bearing areas of three femoral heads with five plugs on each femoral head (i.e., each OA stage) using a biopsy punch. Among those five samples extracted on each femoral head, two samples were used to measure µ and surface roughness (R_q_) at 16 locations in PBS. The remained three samples on each femoral head with a corresponding OA stage were repeatedly used for the measurements of µ and R_q_ in all concentrations of HA and **γ**-globulin; 10 locations from 3 samples on each **γ**-globulin concentration and 16 locations from 3 samples on each HA concentration. To repeat frictional measurements with different concentrations of HA and **γ**-globulin, the used cartilage samples were washed with 1X PBS for 30 min. The test samples were stored in PBS at 4°C for no more than 24 h before experiments. Prior to frictional measurements, each OA cartilage sample was glued to a cylindrical plate (diameter = 19 mm, height = 1 mm), and then affixed to a 50 mm polystyrene petri dish with cyanoacrylate adhesive.

**Table 1:** Outerbridge classification.

Grade pathology	
0	Normal cartilage
I	Softening and swelling of articular cartilage
II	Fragmentation and fissuring of articular cartilage affecting an area of less than 0.5 inches
III	Fragmentation and fissuring of articular cartilage affecting an area greater than 0.5 inches
IV	Cartilage erosion to bone

### Immunohistochemistry

**Figure 2:**
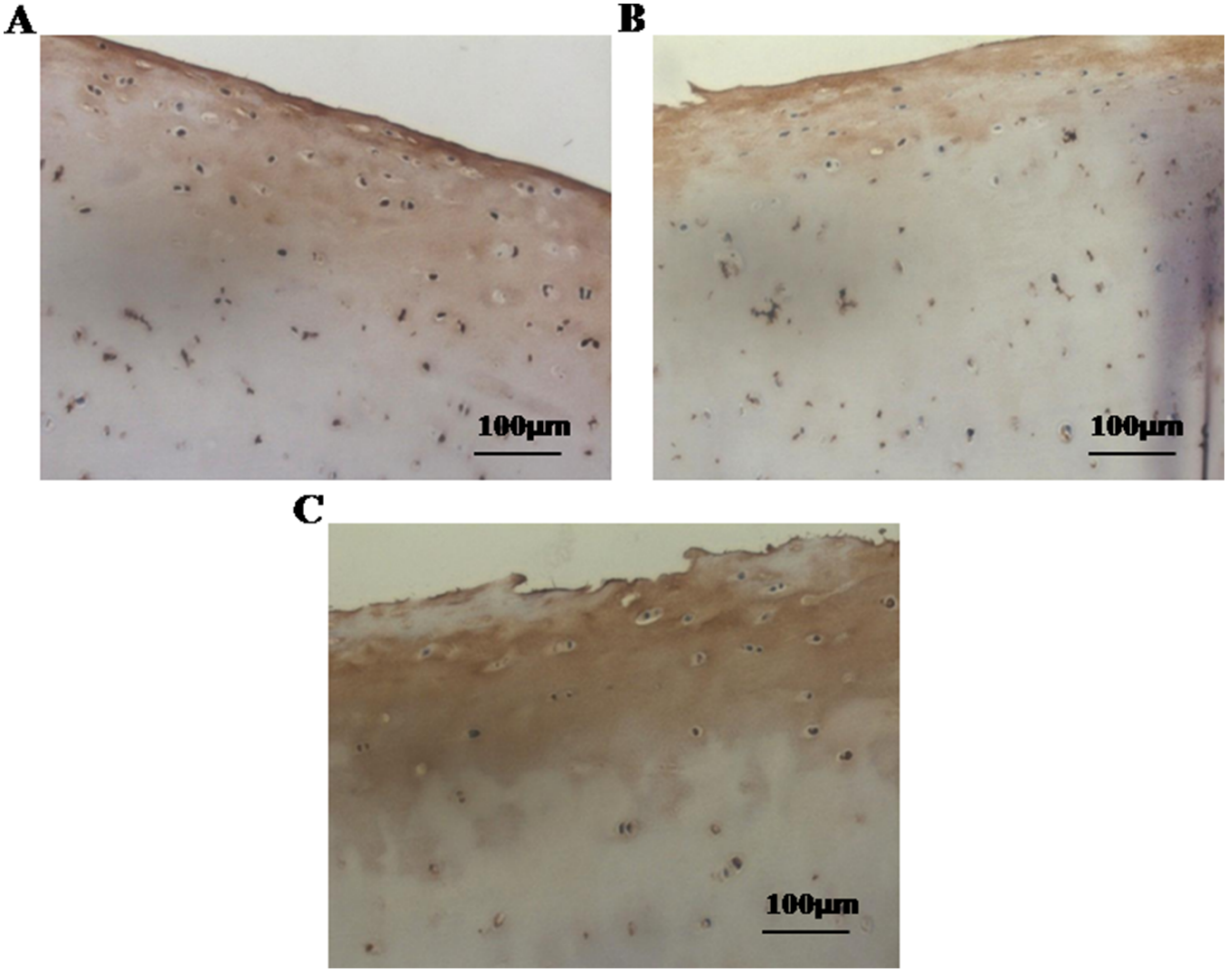
Expression of lubricin by human articular cartilage explants of different OA stages from total hip replacement operations. Immunostaining of lubricin represented decreased expression of lubricin in early (B), and advanced OA cartilage (C) than normal cartilage (A); Magnification x20; scale bars 100 µm.

Random samples from femoral heads of various OA stages were immunostained with the two-step immunohistochemistry method as instructed by the manufacturer. In brief, deparaffinized sections were incubated overnight with rabbit polyclonal antibody against lubricin (sc-98454, Santa Cruz Biotechnology INC., CA, USA), 1∶200 dilution, at 4°C. The slides were washed three times in PBS followed by 30 min incubation at room temperature with goat anti-rabbit immunoglobulin G (IgG) (Santa Cruz Biotechnology INC., CA, USA) and visualized with DAB chromagen. Stained sections were photographed with a Ziess AxioCam digital camera, at 20× magnification with constant illumination intensity (Figure 2).

### Safranin O Staining

**Figure 3:**
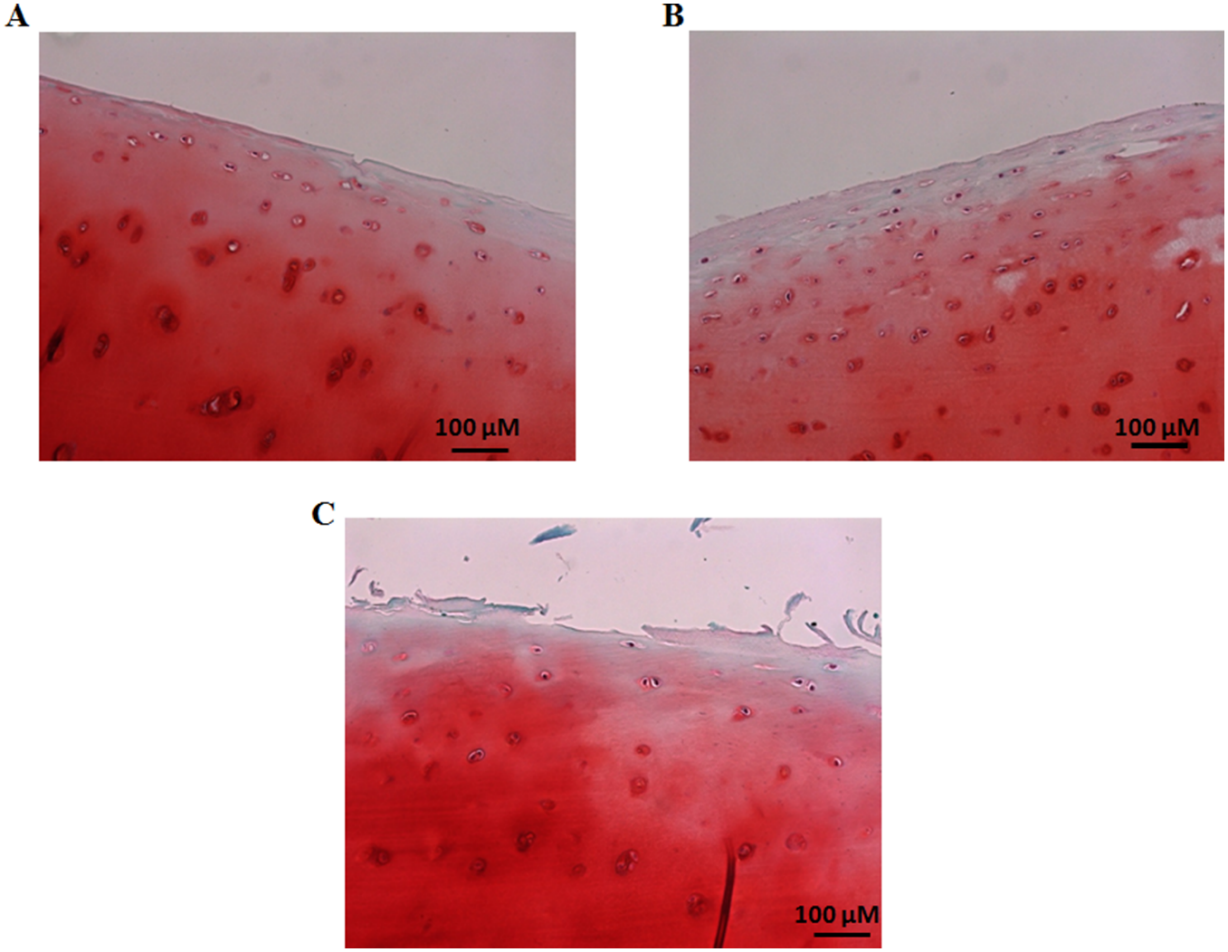
Representative histologic staining with Safranin O fast green. (A) Normal human articular cartilage section shows uniform Safranin O staining intensity till articular surface. (B) Slight reduction in Safranin O staining intensity at the articular surfaces is observed in a cartilage sample with early OA stage (C) reduced Safranin O staining is observed at the articular surface of a cartilage sample with advanced OA stage due to rupture of cartilage. Magnification x20; scale bars 100 µm.

For Safranin O staining, deparaffinized sections were first stained with fast green solution for 5 min. Then slides were quickly rinsed with 1% acetic acid solution for 15 sec. Finally, samples were stained with 0.1% Safranin O solution for 5 min. Thereafter, samples were dehydrated and cleared two times each for 2 min with 95% ethyl alcohol, absolute ethyl alcohol, and xylene. Slides were mounted for taking picture with a Ziess AxioCam digital camera, at 20× magnification (Figure 3).

### Lubricant Preparation

Three lubricants at different concentrations were prepared. PBS (P5493, Sigma–Aldrich, St. Louis, USA) was used as control solution. Bovine γ–globulin (5009, Sigma–Aldrich, St. Louis, USA) was dissolved in PBS at concentrations of 0.5 and 2.0 mg/mL and used as a lubricant; AFM frictional measurements was difficult to perform at higher concentrations than 2.0 mg/mL due to the highly thick viscosity of γ–globulin at those concentrations, although the γ–globulin level in human SF ranges from 1.0 to 4.2 mg/mL [15]. Finally, HA (53747, Sigma–Aldrich, St. Louis, USA) from *Streptococcus equi* with a molecular weight of MW = 1.63 MDa was dissolved in PBS at 1.0, 3.0 and 5.0 mg/mL concentrations by stirring in an ultrasonic bath at 60°C for 3 h. These HA concentrations were preferred based on the physiological concentration of human SF reported to range from 3.0 to 4.0 mg/mL [15].

### AFM Friction Measurements

**Figure 4:**
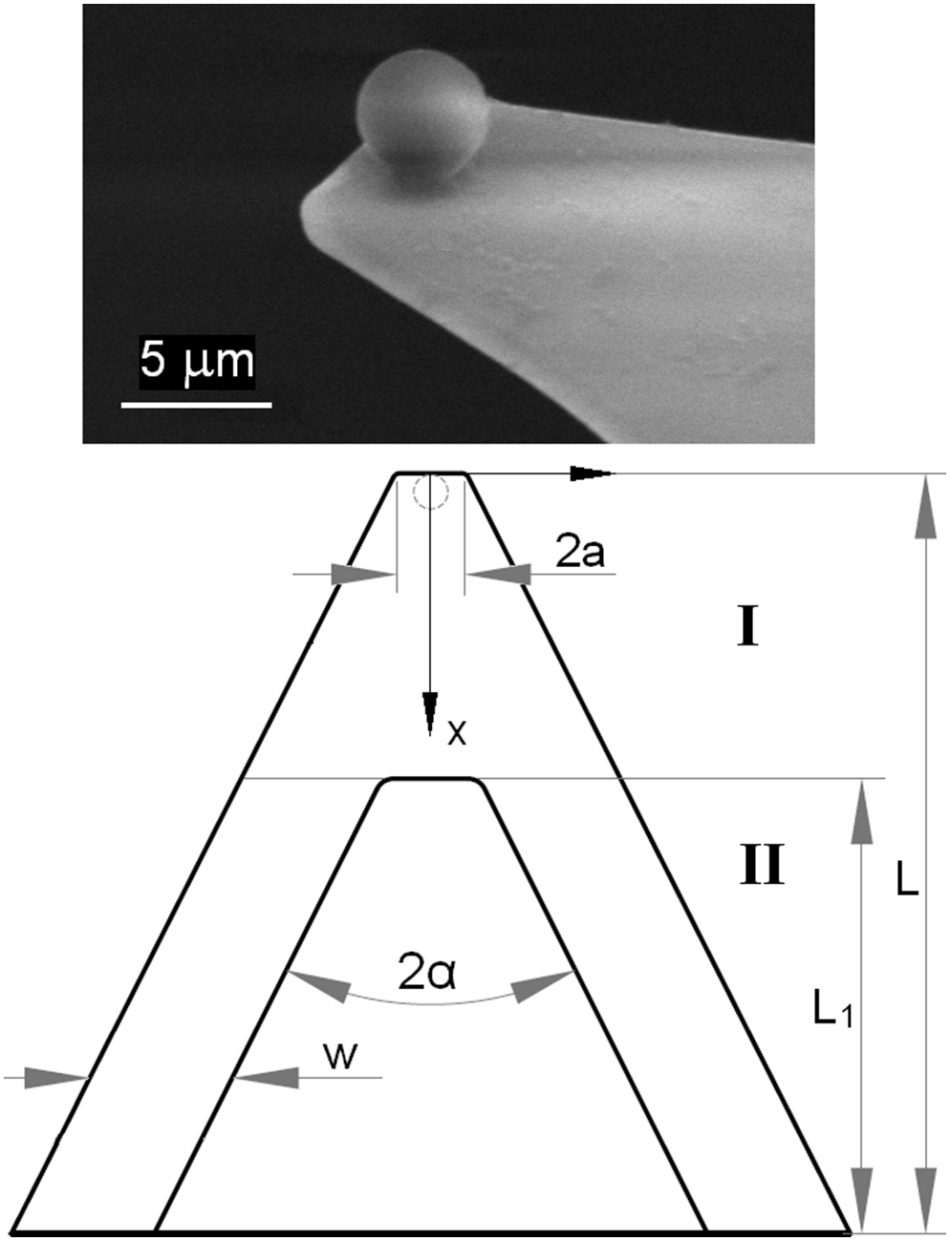
Scanning electron microscopy image of a triangular silicon–nitride cantilever with a polystyrene spherical tip (4.5 µm diameter) attached, and detailed parameters of the cantilever.

**Figure 5:**
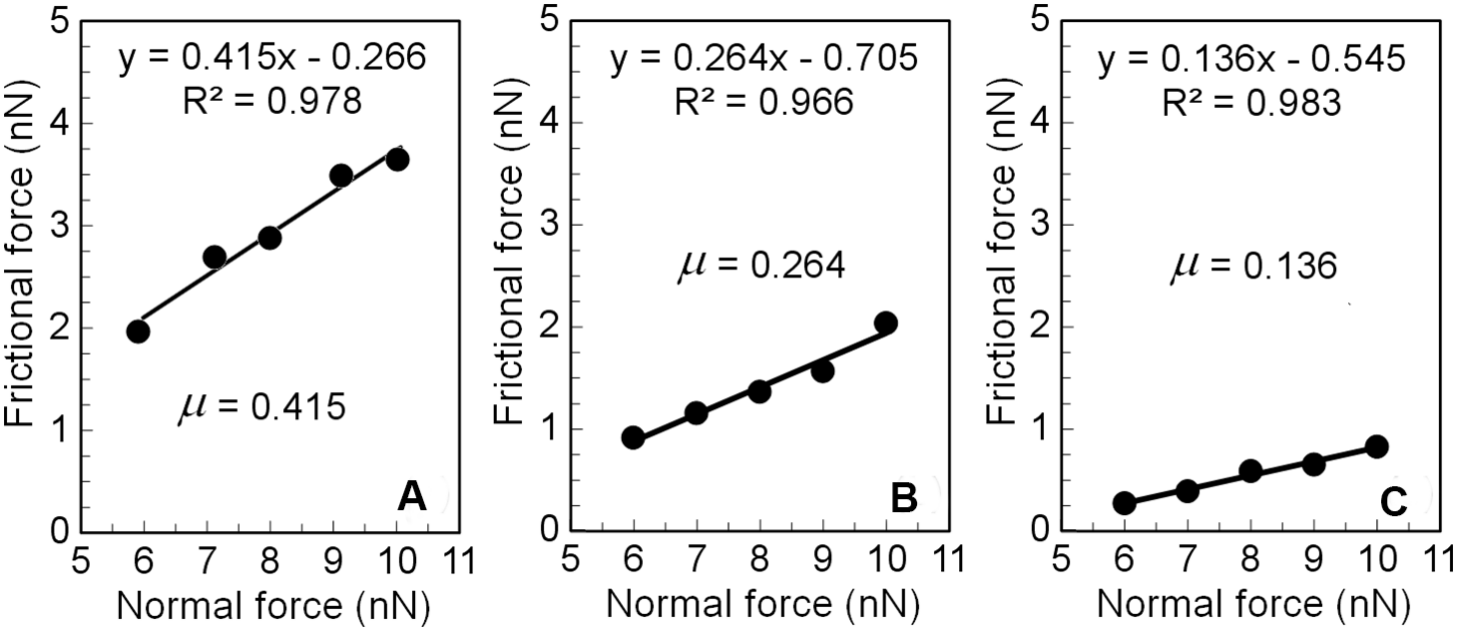
Representative plots of frictional versus normal forces for advanced OA cartilage measured in the lubricants of (A) PBS and, (B) HA 3.0 mg/ml, and (C) γ–globulin 2.0 mg/ml. The linear slope yielding the AFM frictional coefficient measured for five values of the normal force in the range of 6–10 nN at an increment of 1 nN at each location.

**Figure 6:**
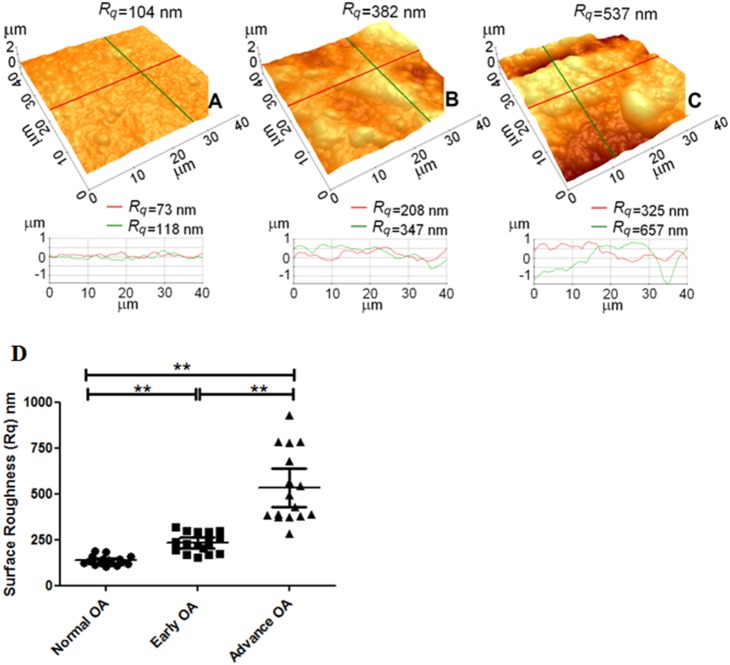
Typical AFM images of human cartilage surfaces with OA and line profile analyses measured in PBS, showing severe wear on the cartilage surface with an advanced stage OA. (A) normal cartilage, (B) early OA cartilage, and (C) advanced OA cartilage. (D) Surface roughness (Rq) of human articular cartilage of normal, early and advanced OA in PBS solution, represents significant increase in Rq value with increasing OA stages. (**P<0.0001). The values of Rq were from 16 different locations on two cartilage samples of each OA score from a single femoral head. Vertical bars represent 95% of confidence interval (no of observation = 16).

The microscale friction and R_q_ of human OA cartilage samples were measured by an AFM (XE 70, Park Systems Corp., South Korea) with the probe of a triangular silicon−nitride (Si_3_N_4_) cantilever (normal spring constant, *k_N_* = 0.35 N/m) and a polystyrene spherical tip of 4.5 µm diameter (Novascan Technologies, Ames, Iowa, USA). Because, AFM measurements were conducted with cartilage samples submerged entirely in lubricant solutions at room temperature (16°C), the AFM probe was equilibrated thermally for 30 min in the lubricants prior to AFM measurements. The microscale µ was calculated from the linear slope of the plot of AFM measured frictional versus the applied normal force, for a 40 µm×40 µm area scanned in contact mode (resolution: 256×256 pixels, scan rate: 1 Hz (80 µm/s). The applied normal force was acquired from the provided data analysis program (XEI, Version 1.8.0, Park Systems Corp., South Korea), while the calculation of frictional force is described in detail in our previous study [20]. Briefly, in order to calculate the frictional force, the values for the lateral voltage signal (*V_LFM_*) of the scanned image, the lateral spring constant (*k_L_*), and the lateral sensitivity (*S_L_*) of the triangular cantilever should be first obtained. *S_L_* was calculated analytically from the normal sensitivity (*S_N_* = 75 nm/V) measured on glass in the current study [29], and *V_LFM_* was computed as one–half of the mean difference between the forward and backward voltage signals measured from the scanned image. Then, *k_L_* was calculated with a new method developed in our recent study [30]. In the study, the cantilever was subdivided into parts I and II as shown in figure 4, and torsion theory was employed to calculate the twist angle (*φ*), which resulted from a constant torque *T* applied to each part of the cantilever, with the equation, 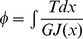, where *J* was the polar moment of inertia, and *G* was the cantilever shear modulus. The torsional spring constant (*k_φ_*) was calculated from *k_φ_* = *T/φ*. Finally, *k_L_* was computed from the following equation, in relation to the normal spring constant [31] (*k_N_* =  *Ewt* ^3^/2*L* ^3^):
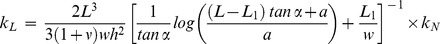
(1)


where *t* is the AFM cantilever thickness, *h* is the AFM tip height, and *ν* = 0.27 is the Poisson’s ratio of the AFM cantilever, with other parameters shown in figure 4. These parameters of the AFM cantilever and tip were measured using an optical microscope (Eclipse LV100, Nikon Instruments, Japan) to calculate eq. (1), and the calculated value of *k_L_* was 110 N/m. The frictional force (*F_L_*) was calculated by the equation, *F_L_ = V_LFM_*×*S_L_*×*k_L_*, for the 40 µm×40 µm scanned area. Then, the microscale frictional coefficient µ were calculated from the linear fit of the friction versus normal force, and representative plots of frictional versus normal forces for advanced OA cartilage measured in the lubricants of PBS and, 3.0 mg/ml HA, and 2.0 mg/ml γ–globulin are also shown in figure 5. Mean ± standard deviation of the microscale frictional coefficient µ and correlation coefficient (*R^2^*) were calculated for different cartilage surfaces with different OA stages as well as different lubricant concentrations (Table 2). The root mean squared values of surface roughness (R_q_) were simultaneously obtained over the same area of AFM frictional measurements (Figure 6).

**Table 2:** Mean ± standard deviation (SD) of the microscale frictional coefficient (*µ*) and correlation coefficient (*R^2^*) for human OA cartilage measured in PBS, HA, and γ–globulin.

Lubricant	Concentration	*µ* ± SD (*R^2^* ± SD, n = measurements number)		
		Normal	Early OA	Advanced OA
**PBS**		0.119±0.036 (0.973±0.031, n = 16)	0.151±0.039 (0.976±0.040, n = 16)	0.409±0.119 (0.976±0.056, n = 16)
**HA**	1.0 mg/ml	0.126±0.038 (0.928±0.059, n = 16)	0.160±0.059 (0.907±0.056, n = 16)	0.262±0.083 (0.959±0.060, n = 16)
	3.0 mg/ml	0.119±0.027 (0.976±0.017, n = 16)	0.152±0.059 (0.941±0.038, n = 16)	0.269±0.119 (0.981±0.017, n = 16)
	5.0 mg/ml	0.181±0.039 (0.971±0.017, n = 16)	0.143±0.041 (0.940±0.038, n = 16)	0.221±0.067 (0.914±0.074, n = 16)
**γ–globulin**	0.5 mg/ml	0.207±0.042 (0.962±0.022, n = 10)	0.203±0.050 (0.979±0.011, n = 10)	0.266±0.089 (0.962±0.038, n = 10)
	2.0 mg/ml	0.182±0.055 (0.932±0.031, n = 10)	0.213±0.053 (0.898±0.073, n = 10)	0.126±0.039 (0.915±0.069, n = 10)

### Statistical Analysis

All values for R_q_ and µ were tested with the Kolmogorov-Sminov test for normal distribution and the Levine’s test for variance. Statistical analysis of the data was performed using the Kruskal-Wallis test (two-sided) and one-way analysis of variance (ANOVA) to detect significant differences in the R_q_ between the different OA stages and in the microscale µ of OA cartilage between PBS and different concentrations of HA, as well as between PBS and different concentrations of γ–globulin (SAS 9.4, SAS Institute Inc., Cary, NC, USA). Data is reported as mean with 95% confidence intervals.

## Results

### Characterization of cartilage surface from human femoral head of normal, early and advanced OA stages

Prior to analysis of frictional behavioral studies, site-specific surface irregularities of samples were determined by histological staining (lubricin and Safranin O staining). Lubricin is synthesized by synovial and cartilage cells and plays an important role in joint lubrication [32]. Immunohistochemistry of lubricin showed decreased expression of lubricin at the articular surface of human femoral head cartilage with early and advanced OA stages than normal (Figure 2). Safranin O staining is known to bind glycosaminoglycan of articular cartilage. Our samples displayed reduced color intensity in early and advance stages compared to normal, indicating stage dependent nature of our samples (Figure 3).

### Characterization of cartilage R_q_ of OA stages using AFM

To further characterize the surfaces of cartilages utilized for frictional studies, their R_q_ was analyzed with AFM. Figure 6 shows AFM topography images of the cartilage samples measured in PBS with normal (A), early (B) and advance (C) OA stages. The mean R_q_ values increased significantly with increasing OA stage: R_q_ = 137±25 nm (n = 16) for the normal cartilage surface, 233±55 nm (n = 16) for the early OA cartilage surface and 533±196 nm (n = 16) for the advanced OA cartilage surface. Significant differences in R_q_ were observed among the cartilages of different OA stages (between normal and early OA; between normal and advanced OA; between early OA and advanced OA, **P<0.0001) (Figure 6(D)).

### Effect of HA and γ–globulin on µ of normal cartilage of human femoral head

**Figure 7:**
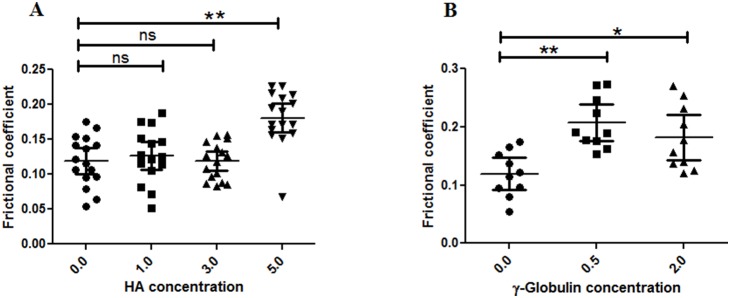
AFM measured frictional coefficient of normal human articular cartilage in range of (A) HA (no of observation = 16) and (B) γ–globulin (no of observation = 10) concentration. Increase in HA concentrations shows no significant changes in frictional coefficient of normal articular cartilage samples, while γ–globulin depicts concentration based effect. The frictional coefficient values are from measurements at different locations on two samples of normal human cartilage from a single femoral head. Vertical bars represent 95% of confidence interval. *P<0.01, **P<0.0001, and ns (no significance).

For normal cartilage, none of the HA and γ–globulin concentrations improved cartilage frictional response (Figure 7, Table 2). On the contrary, µ exhibited a significantly higher value at the 5.0 mg/mL HA concentration group (µ = 0.181±0.039) than at the PBS group and other HA concentration groups (Figure 7(A)) and increased with increasing γ–globulin concentration (between 5.0 mg/ml HA and PBS, *p*<0.0001; between 0.5 mg/ml γ–globulin and PBS, *p*<0.0001; between 2.0 mg/ml γ–globulin and PBS, *p* = 0.002) (Figure 7(B)).

### Effect of HA and γ–globulin on µ of cartilage of early OA Stage

**Figure 8:**
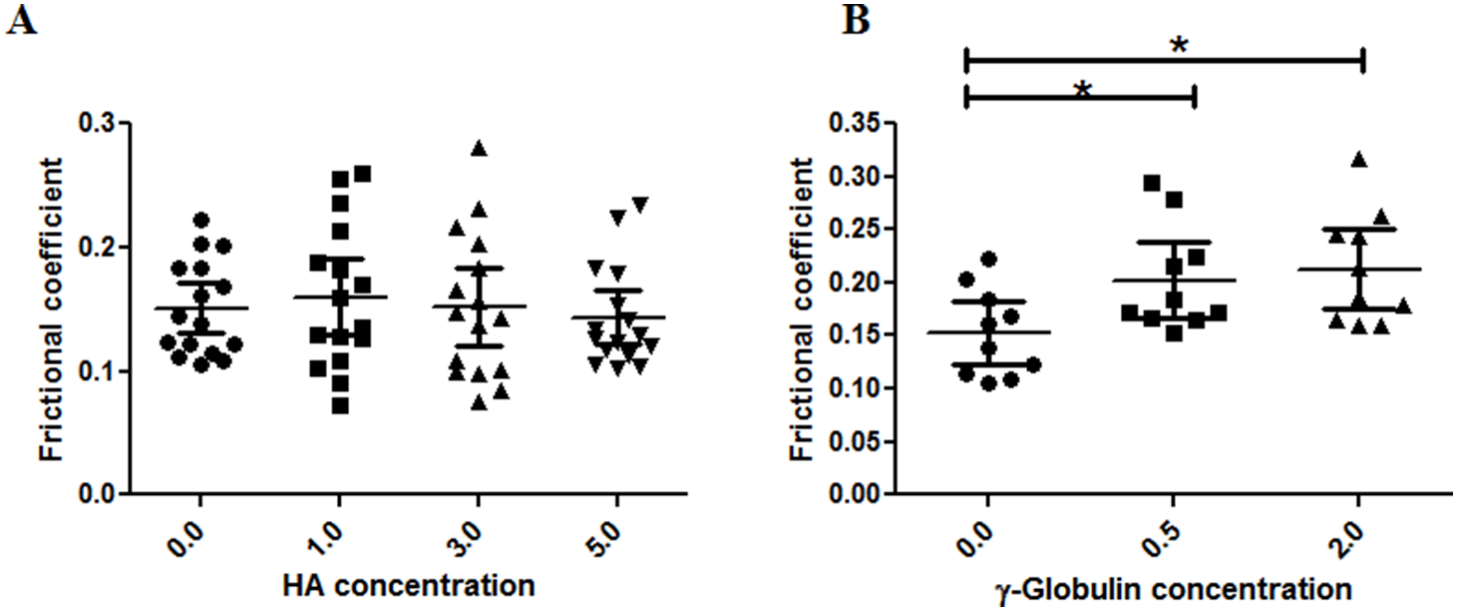
AFM measured frictional coefficients of early OA cartilage measured in a range of (A) HA (no of observation = 16) and (B) γ–globulin (no of observation = 10) concentrations. In case of HA, no significant changes in frictional coefficient were observed. γ–globulin shows concentration based effect on frictional coefficient. The frictional coefficient values are from measurements at different locations on two samples of normal human cartilage from a single femoral head. Vertical bars represent the 95% of confidence interval. *P<0.01.

For early OA cartilage, the concentration–dependent lubricating ability of HA and γ–globulin was similar to that for normal cartilage (Figure 8, Table 2). No significant differences in µ were observed between the PBS group and any of the HA concentration groups (Figure 8(A)), whereas the µ of the 0.5 and 2.0 mg/mL γ–globulin concentration groups were higher than the µ of the PBS group (between 0.5 mg/mL γ–globulin and PBS, *p* = 0.007; between 2.0 mg/mL γ–globulin and PBS, *p* = 0.002) (Figure 8(B)).

### Effect of HA and γ–globulin on µ of cartilage of advanced OA Stage

**Figure 9:**
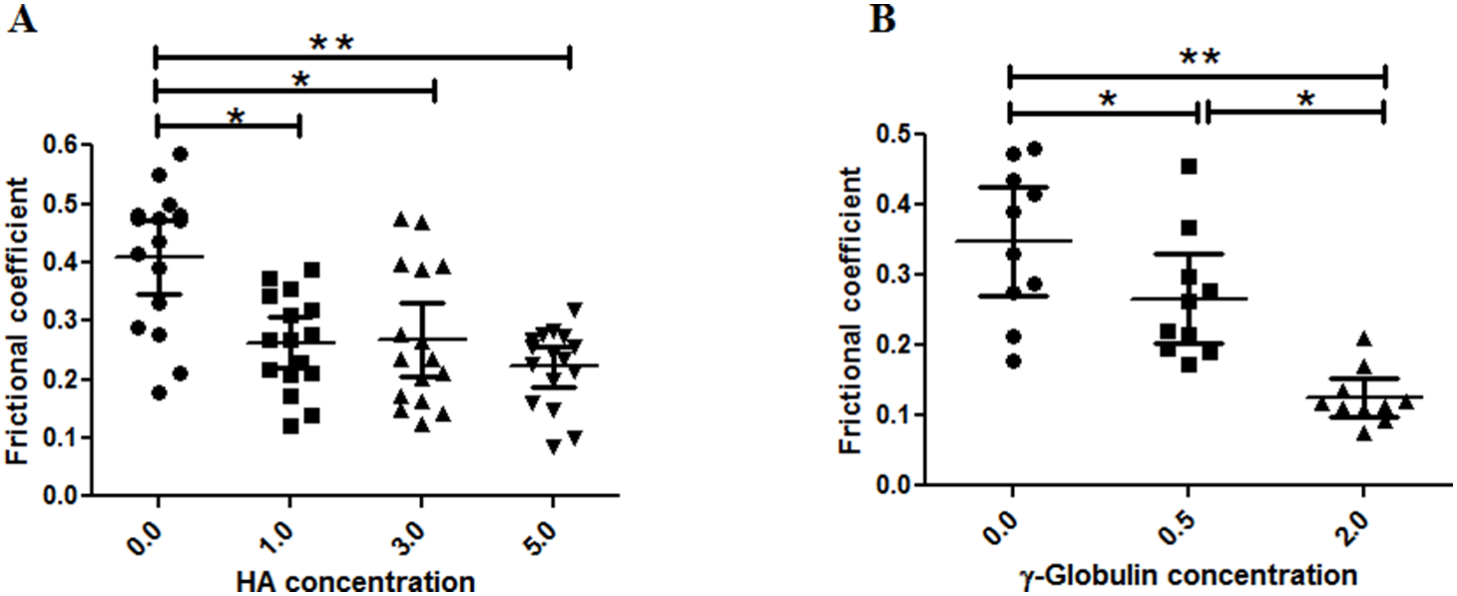
AFM measured frictional coefficients of advanced OA cartilage measured in a range of (A) HA (no of observation = 16) and (B) γ–globulin (no of observation = 10) concentrations. Increased concentration of HA and γ–globulin shows a decrease in frictional coefficients of advanced OA cartilage samples. The frictional coefficient values were from measurements at different locations on two samples of advanced human cartilage from a single femoral head. Vertical bars represent the 95% of confidence interval. *P<0.01 and **P<0.0001.

In advanced stage OA, both HA and γ–globulin improved cartilage frictional behavior significantly (Figure 9, Table 2). Improvement in cartilage friction by HA was not concentration–dependent, as shown by the result that the µ of the PBS group was statistically higher than those of the HA concentration groups (between PBS and 1.0 mg/mL HA, *p* = 0.0004; between PBS and 3.0 mg/mL HA, *p* = 0.003; between PBS and 5.0 mg/mL HA, *p*<0.0001), while no significant difference was detected between any of the HA concentration groups (Figure 9(A)). However, cartilage lubrication in advanced-stage OA was enhanced with γ–globulin in a concentration-dependent manner, showing that µ decreased with increasing γ–globulin concentration (between 0.5 mg/ml γ–globulin and PBS, *p* = 0.003; between 2.0 mg/ml γ–globulin and PBS, *p*<0.0001; between 0.5 mg/ml and 2.0 mg/ml γ–globulin, *p* = 0.0002) (Figure 9(B)).

### Correlations between R_q_ and µ based on lubricant concentration

**Figure 10:**
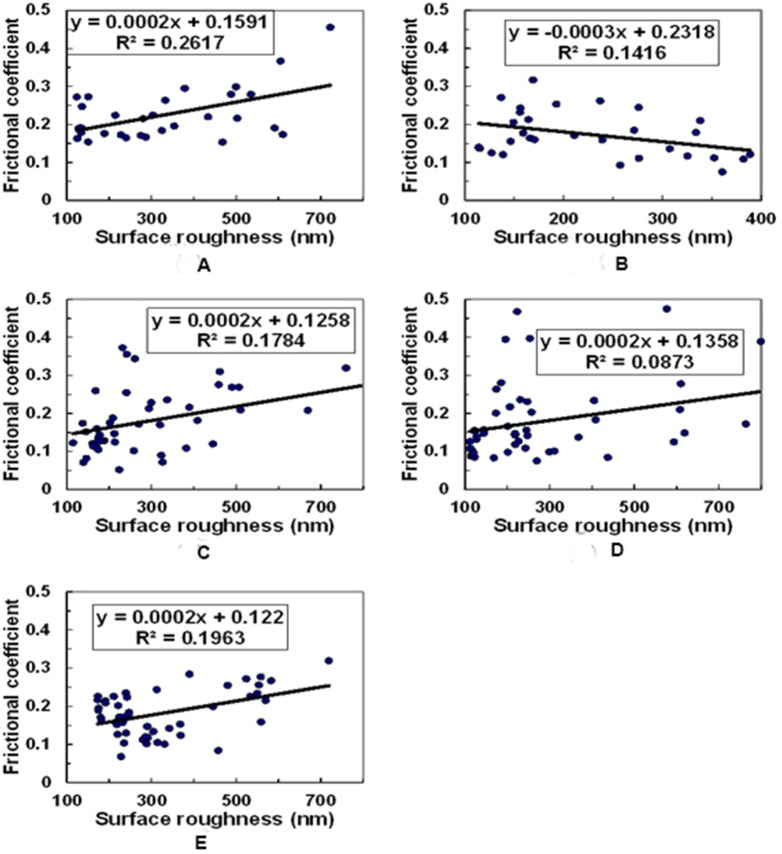
The correlations between surface roughness (R_q_) and frictional coefficient (µ) for (A) 0.5 mg γ-Globulin (n = 30), (B) 2 mg γ-Globulin (n = 30), (C) 1 mg HA (n = 48), (D) 3 mg HA (n = 48), and (E) 5 mg HA (n = 48).

Furthermore, in order to verify the possibility, whether the observed changes in µ with different lubricants were either solely due to variations in surface roughness of specimens, or there is any role of structural changes due to proteoglycan depletion as observed during OA progression, we evaluated the correlations (R^2^) between R_q_ and µ based on lubricant concentration. For this, results from all samples were pooled together and a correlation between R_q_ and µ based on lubricant concentration were analyzed. No significant correlations were observed in any of the cases; the correlations between R_q_ and µ were R^2^ = 0.262 for 0.5 mg γ-Globulin (n = 30) (Figure 10(A)), R^2^ = 0.142 for 2 mg γ-Globulin (n = 30) (Figure 10(B)), R^2^ = 0.178 for 1 mg HA (n = 48) (Figure 10(C)), R^2^ = 0.087 for 3 mg HA (n = 48) (Figure 10(D)), R^2^ = 0.196 for 5 mg HA (n = 48) (Figure 10(E)). Moreover, after adding HA and γ–globulin, correlations between R_q_ and µ were not significant either for normal cartilage (R^2^ = 0.066): early OA cartilage (R^2^ = 0.0008) and advanced OA cartilage (R^2^ = 0.549).

## Discussion

In this study, AFM was used to examine the role of HA and γ–globulin concentrations in the boundary lubricating ability of OA cartilage surfaces in human femoral heads. The hypothesis that HA and γ–globulin would reduce cartilage friction was partly accepted, because HA and γ–globulin were not effective in improving the boundary lubricating ability of both normal (Figure 7) and early OA (Figure 8) cartilage, while, for advanced OA cartilage, both HA and γ–globulin improved cartilage frictional behavior significantly (Figure 9).

In advanced stage OA, the cartilage superficial layer was completely removed (Figure 2 and 3) and the damaged cartilage surface (Figure 6 (A, B and C)) showed a higher µ value (∼0.409) than the normal cartilage surface (∼0.119) in PBS (Figures 7–9). In advanced OA stage, adsorbed HA and γ–globulin molecules demonstrated improved cartilage frictional response (Figure 9). These results were consistent with previous macroscale studies reporting that HA [10], [17] and γ–globulin [33], [34] lubricants could help improve the frictional response of damaged articular cartilage. In advanced OA cartilage, although both HA and γ–globulin played important roles in enhancing cartilage frictional behavior, only γ–globulin showed concentration–dependent improvements in the cartilage frictional response (Figure 9). Results obtained are consistent with the literature findings where no difference in reducing friction of OA cartilage was observed between the low and high concentration (5 and 10 mg/mL) of HA [10], and cartilage µ were reduced by HA only in a molecular-weight dependent manner [35]. Therefore, the concentration or viscosity of HA might not be an important factor in enhancing frictional response of OA cartilage. Thus, it can be suggested that patients with advanced OA are less likely to benefit from intra-articular injection of HA [36]. However, contradictory study also exists, demonstrating dose-dependent effect of HA in reducing µ for fresh bovine osteochondral samples [37].

For the normal cartilage surface, the use of HA and γ–globulin, as lubricants, did not improve the frictional response (Figure 7). In agreement to this result, CJ bell et. al., also demonstrated that the addition of HA to the normal human articular cartilage produced no significant differences at any time point under dynamic friction tests [17]. Moreover, the finding that cartilage frictional behavior was better in normal cartilage than advanced OA cartilage (Figure 7–9), as confirmed by figure 2, might be due to the presence of more lubricin in normal cartilage. It has been reported that boundary-lubricating ability of cartilage is reduced with a decrease in PRG4 (proteoglycan 4, also known as lubricin) [38]. Furthermore, our results demonstrating improved frictional behavior of cartilage only in advanced OA samples by HA and γ–globulin compared to normal (Figure 8) might suggest that HA and γ–globulin are unable to impart any further additive improvement to normal cartilage with lubricin. It is possible that lubricin or proteoglycan in the cartilage superficial layer itself is enough to provide excellent lubricating ability. However, these results were somewhat unexpected based on some earlier studies reporting synergetic improvement of cartilage friction by HA and lubricin [35], [37], while another study reported contradictory results that cartilage frictional coefficient increased with decreasing HA and increasing PRG4 [39]. Even more surprisingly, the use of γ–globulin as a lubricant made the cartilage frictional behavior worse (Figure 7(B)), presumably due to a possible adverse interaction between γ–globulin and some components (or component aggregates) of the cartilage superficial layer which needs to be investigated by future studies. Moreover, although the effects of HA molecular weight on the friction response of articular cartilage were not investigated in the current study, Kwiecinski et al. reported that cartilage frictional coefficients were reduced with an increase in HA molecular weight, in a molecular-weight dependent manner [35]. It was supported by Antonacci et al. who reported that the cartilage friction coefficient of equine SF from normal joints was lower than that of equine SF from joints with acute injury that had a lower HA molecular weight [39]. However, the contradictory result that cartilage frictional coefficient increased with increasing HA molecular weight was also observed after intra-articular injections of HA into rabbit knee joints [40], while the frictional behavior of rabbit knee joints, measured by a robotic arm under force-control, was not dependent on HA molecular weight [41].

In early-stage OA, the surface of articular cartilage is partially damaged, yet, use of HA and γ–globulin at physiological concentration levels as lubricants did not improve the frictional response. When measured in PBS, the µ of early OA cartilage (∼0.151) was higher than that of normal cartilage (∼0.119) (Figure 8). As described above, some proteoglycans remaining on the cartilage surface in early-stage OA, probably due to the relatively well preserved cartilage superficial layer, may still impart lubricating ability to the cartilage surface. This study used the physiological concentration of human SF with concentration range of 1–5 mg/mL of HA. Nevertheless, practically a higher visco-supplementation with HA can be supplied with elevated concentrations and molecular weight in OA joints [39], [42]. Caligaris et al. studied the frictional behavior of human arthritic knee cartilage and reported that use of bovine SF decreased the frictional coefficient of the osteoarthritic tibiofemoral joint in both early and advanced OA stages, relative to PBS [43]. In the present study, however, the effects of two main constituents of SF (HA and γ–globulin) and their diverse concentrations on the friction of the osteoarthritic hip joint were investigated. Our results demonstrate that both the components in SF displayed no significant role in reducing the cartilage frictional coefficient in early-stage OA. However, lowering of frictional coefficient by SF in arthritic knee joints, as observed by Caligaris et al., might by suggesting that the other two main components in SF, i.e. serum albumin and dipalmitoyl phosphatidylcholine (DPPC), could be involved in reducing the frictional coefficient of early-stage OA cartilage. Moreover, possibilities of disease modifying effect by HA and γ–globulin in OA cartilage cannot be ruled out and further studies are needed to explain the current results observed [44].

Clinically, a detailed tribological evaluation can give helpful information to clarify the mechanism of symptomatic improvement with intra-articular HA injection in early OA patient. But, due to the limitations in obtaining the human cartilage samples with early OA, only few studies for early stage OA on friction tests with HA have been reported [10]. To obtain a normal femoral head for comparative cartilage study is not always possible and thus current study suffers from the limitation of not more than one femoral head per each OA stage. However, to examine whether the observed results are truly general or just a chance outcome from normal biological variability, we analyzed all the values for surface roughness and frictional coefficient with the Kolmogorov-Sminov test for normal distribution and the Levine’s test for variance, with the help of a statistician from our institute. Through these tests, all the values in each group had proved to show a normal distribution pattern, suggesting that each group of sample deserve to be used for the experiments.

Furthermore, from a clinical perspective, both initial and steady state frictional coefficients are necessary to fully understand the contribution of boundary lubricants in the frictional response of articular cartilage. For example, by observing the initial minimum frictional coefficient that is dependent on interstitial fluid pressurization in cartilage, cartilage tissue engineering research can focus on minimizing loss of interstitial fluid pressurization in cartilage. On the other hand, by investigating AFM frictional coefficient that is more representative of the steady state equilibrium frictional coefficient (the steady state equilibrium frictional coefficient represents the intrinsic material property of the solid matrix in the absence of interstitial fluid pressurization, and hence, effective boundary lubricants would help reduce the value of steady state frictional coefficient), efforts in the development of clinical treatment modalities can focus on delivering the most effective boundary lubricant onto the articular surfaces. Therefore, although being unable to measure the initial frictional coefficient by AFM is a limitation in the current study, we believe that AFM friction measurements would be ideal for investigating the effectiveness of putative boundary lubricants on the cartilage frictional response.

## Conclusion

HA and γ–globulin significantly enhanced cartilage friction only in advanced-stage OA, but not in normal and early-stage OA. Improvement in the frictional behavior of advanced OA cartilage was concentration-dependent with γ–globulin, whose concentration within the physiological range provided the best cartilage lubrication. Although these differences in the cartilage frictional behavior by HA and γ–globulin lubricants were discussed in relation to proteoglycans changed with OA progression, it needs to be further quantitatively investigated, thereby contributing to determining the clinical treatment timing and application dose of these lubricants for patients with OA.
