# Correction: Effects of Hyaluronic Acid and γ-Globulin Concentrations on the Frictional Response of Human Osteoarthritic Articular Cartilage

**DOI:** 10.1371/journal.pone.0118040

**Published:** 2015-02-10

**Authors:** The PLOS ONE Staff

The Funding section of the published article should read: This research was supported by Basic Science Research Program through the National Research Foundation of Korea (NRF) funded by the Ministry of Education, (NRF-2013R1A1A2062436), by Leading Foreign Research Institute Recruitment Program through the NRF grant funded by the Ministry of Science, ICT & Future Planning (No.2009–00495), and by the Korea Health Technology R&D Project through the Korea Health Industry Development Institute (KHIDI), funded by the Ministry of Health & Welfare, Republic of Korea (HI12C1265). The funders had no role in study design, data collection and analysis, decision to publish, or preparation of the manuscript.
